# Directed Acyclic Graphs for Causal Modeling of Musical Interventions in High-Tropical Grazing Dairy Cattle: A Causal Framework Review

**DOI:** 10.3390/vetsci13101040

**Published:** 2026-10-02

**Authors:** Patricia Betancourth-Chaves, John Jairo Montoya-Zuluaga, Berardo de Jesús Rodríguez, José Eduardo Escobar-Riomalo, Darío Antonio Vallejo-Timarán

**Affiliations:** 1 Research Group Patobiología Quirón, School of Veterinary Medicine, Agrarian Sciences Faculty, University of Antioquia, Medellín 050036, Colombia; patricia.chaves@uam.edu.co (P.B.-C.); berardo.rodriguez@udea.edu.co (B.d.J.R.); 2 Research Group GIsCA, Faculty of Veterinary Medicine and Animal Sciences, Institución Universitaria Visión de las Américas, Medellín 050031, Colombia; john.montoya@uam.edu.co (J.J.M.-Z.); jose.escobar@uam.edu.co (J.E.E.-R.); 3 Obonuco Research and Technology Transfer Center, Corporación Colombiana de Investigación Agropecuaria–AGROSAVIA, Pasto 520038, Colombia

**Keywords:** causal inference, grazing dairy cattle, biomusicology, animal behavior

## Abstract

Musical exposure in dairy cattle has been associated with potential effects on milk production, behavior, and physiological regulation; however, available evidence remains heterogeneous and potentially affected by biological and environmental confounding. In high-tropical grazing systems, these responses are simultaneously influenced by stress physiology, disease, nutrition, management, climatic variability, and individual adaptation. This review integrated causal inference and graphical epidemiology to develop biologically grounded directed acyclic graphs (DAGs) representing hypothesized causal pathways between musical exposure and milk production, behavior, and heart rate variability (HRV). An analytical narrative synthesis of physiological, ethological, autonomic, and epidemiological evidence was conducted, and three independent DAGs were constructed using DAGitty version 3.1. Variables were classified according to their epidemiological roles. The production DAG identified stress as a major integrative node linking disease, nutrition, reproductive physiology, management, and milk yield. The behavioral DAG highlighted the roles of ecological organization, resource access, social interaction, and environmental instability. The HRV DAG treated HRV as beat-to-beat variation in cardiac intervals that provides a non-invasive index of autonomic nervous system (ANS) modulation, including sympathetic and parasympathetic influences, and indicated that responses may occur through indirect modulation of stress physiology and vagal activity. We conclude that musical interventions should be interpreted as context-dependent sensory exposures rather than isolated causal determinants; the proposed DAGs provide an explicit basis for selecting covariates, avoiding inappropriate adjustment, and designing future studies of dairy cattle welfare and production.

## Simple Summary

Music has been proposed as a potential environmental enrichment strategy in dairy cattle systems; however, its effects on milk production, animal behavior and physiological regulation remain inconsistent across studies. One reason for these inconsistencies is that cattle responses are influenced simultaneously by stress, disease, nutrition, environmental conditions, management and physiological stage. In this review, we used directed acyclic graphs to organize and visualize the complex causal relationships linking musical exposure with milk production, behavior and heart rate variability in high-tropical grazing dairy cattle systems. Three independent DAGs were developed to represent the biological and epidemiological pathways potentially involved in each outcome. The three outcomes were selected a priori because they represent complementary levels of response: milk production as a distal productive endpoint, behavior as an observable welfare and adaptation endpoint, and HRV as a rapid physiological endpoint of autonomic regulation. Separate DAGs were used because each outcome has distinct causal antecedents, mediators, and potential confounding structures that would be obscured in a single combined graph. The proposed framework highlights that music should not be interpreted as an isolated stimulus, but rather as a contextual factor interacting with stress physiology, environmental conditions and animal adaptation. This approach may help improve future study design, variable selection and interpretation of acoustic interventions in dairy cattle welfare and production research.

## 1. Introduction

Studies evaluating musical exposure in dairy cattle have produced heterogeneous and sometimes contradictory findings regarding milk production, behavior, and physiological regulation. Recent studies have reported changes in milk letdown, residual milk, welfare-related indicators, or harvested milk under specific musical protocols, whereas other behavioral and milking parameters were unaffected, emphasizing strong dependence on stimulus characteristics and study context [1,2]. Within biomusicology, defined as “the interdisciplinary study of the biological foundations, evolution, perception, and effects of music, including comparative perspectives on animal communication” [3], acoustic interventions can be examined as structured sensory signals with potential neurophysiological and behavioral consequences. In this review, “music exposure” or “musical exposure” is operationally defined as the intentional presentation of structured, temporally organized acoustic material with identifiable musical attributes (e.g., rhythm, tempo, timbre, and spectral content), delivered under specified conditions of sound level, duration, timing, location, and distance from the animals; uncontrolled background farm noise and incidental non-musical sounds are not considered music exposure. Because simplified analyses may inadequately account for the causal network linking environment, management, physiology, stress, and production, the interpretation of musical interventions remains biologically and epidemiologically uncertain.

In dairy production systems, the response to an acoustic stimulus occurs within a biologically interconnected network involving metabolic status, disease occurrence, environmental stressors, nutritional dynamics, social interactions, lactation stage, and milking management [4,5,6,7,8,9]. Consequently, evaluating the effect of music only through crude changes in milk yield or isolated behavioral observations can lead to confounding, overadjustment, or biologically implausible interpretations. Variables such as stress, disease, feeding behavior, thermal load, and physiological transition may simultaneously act as confounders, mediators, or effect modifiers depending on the causal structure under investigation. In high-tropical grazing dairy systems, causal interpretation becomes even more challenging because environmental variability strongly influences animal physiology and behavior [4,5,6,7,8]. Factors such as high radiation, daily temperature fluctuations, relative humidity, topography, the distance between the pasture and the milking room, and fluctuations in forage quality simultaneously affect energy balance, autonomic activity and behavior. These interacting conditions create a highly dynamic environment in which the effects of musical exposure may be easily confounded by broader ecological and management-related factors.

In this context, directed acyclic graphs (DAGs) provide a useful framework for explicitly representing causal assumptions and identifying the structural relationships linking exposure, physiology, management, and outcomes. DAG-based approaches allow us to distinguish confounding pathways from mediating mechanisms, avoid inappropriate statistical adjustment, and define minimal sufficient adjustment sets for estimating total causal effects. These principles have been extensively described in epidemiology and causal inference research [10,11,12,13] and are increasingly relevant for complex biological systems such as dairy production environments. When applied to cattle production medicine, DAGs are particularly valuable because they require a transparent representation of which variables are considered structural antecedents of the exposure, which are plausible intermediate pathways, and which could become confounders if adjusted incorrectly.

Despite increasing interest in environmental enrichment and acoustic modulation in animal production systems, direct bovine evidence remains limited and heterogeneous [1,2]. Reported responses vary with the acoustic stimulus, exposure protocol, timing, and management context, and few studies integrate productive, behavioral, and autonomic endpoints within the same causal framework. Environmental and physiological evidence further indicates that thermal load, health status, and stress can modify intake, metabolism, behavior, milk composition, and welfare [4,5,6,7,8,9], while reproductive structure and transition physiology condition vulnerability to additional stressors [14,15]. Accordingly, a causal framework is needed to distinguish effects plausibly attributable to music from those arising through broader biological and management pathways. Therefore, the aim of this review was to develop a biologically grounded causal inference framework using directed acyclic graphs (DAGs) to evaluate the potential effects of musical interventions on milk production, behavior, and heart rate variability in high-tropical grazing dairy cattle systems.

## 2. Methodological Strategy

This review was structured as an analytical narrative synthesis guided by principles of causal inference [10,11,12,13]. The evidence was organized around three prespecified outcome domains because they represent different levels and time scales of response to acoustic exposure: (1) milk production, a distal productive outcome influenced by neuroendocrine milk ejection, energy balance, health, and lactation physiology [1,2,14,15,16,17,18,19]; (2) behavior, an observable welfare and adaptation outcome encompassing feeding, rumination, posture, locomotion, social interaction, and resource use within grazing systems [9,20,21,22]; and (3) HRV, a beat-to-beat cardiac measure used as a non-invasive indicator of autonomic modulation and stress responsiveness [23,24,25,26]. Separate DAGs were developed for each domain because the relevant confounders, mediators, effect modifiers, and temporal ordering differ by outcome; combining all outcomes in a single graph would obscure these outcome-specific causal assumptions.

Database searches were performed in PubMed, Scopus, Web of Science, and Google Scholar from January to March 2026; this interval refers to when the searches were conducted, not to the publication dates of eligible studies. No lower publication-date limit was imposed, and literature available through 31 March 2026 was considered. The search strategy combined terms related to dairy cattle, music exposure, acoustic stimulation, stress physiology, heart rate variability, animal behavior, welfare, tropical production systems, and directed acyclic graphs. Search combinations included expressions such as “dairy cattle AND music AND stress”, “musical exposure AND milk production”, “heart rate variability AND dairy cows”, “animal welfare AND acoustic environment”, “heat stress AND dairy cattle”, and “directed acyclic graphs AND veterinary epidemiology”.

Eligibility was based on conceptual relevance rather than a systematic-review screening framework. Inclusion criteria were: (i) peer-reviewed full-text studies in dairy cattle or physiologically comparable livestock/mammalian models addressing music or acoustic stimulation, stress physiology, productive performance, behavior or welfare, HRV, or environmental modulation; (ii) non-bovine mammalian studies only when they directly informed autonomic or neuroendocrine mechanisms not adequately documented in cattle; and (iii) foundational causal-inference and DAG sources required for graph construction. Exclusion criteria were duplicate records; conference abstracts, editorials, or non-peer-reviewed opinion pieces; studies without a clear connection to the exposure, outcomes, or causal variables represented in the DAGs; and reports lacking sufficient methodological or biological information to support a causal link. Because this was an analytical narrative review rather than a systematic review, no PRISMA-style validation stage or pooled effect estimate was performed. The final synthesis comprised 30 sources: 28 peer-reviewed journal articles and two foundational causal-inference books.

Within each domain, the literature was integrated to identify plausible causal pathways, distinguish structural determinants from mediators and effect modifiers, and define outcome-specific causal structures suitable for DAG-based interpretation [10,11,12,13]. References were retained according to biological plausibility, methodological relevance, epidemiological contribution, and applicability to high-tropical grazing dairy systems.

## 3. DAG Development and Causal Assumptions

### 3.1. DAG Construction Basis

The DAGs were constructed and checked in DAGitty version 3.1, following established principles of causal inference and graphical epidemiology [10,11,12,13]. The construction of the DAGs was based on four methodological principles. Firstly, each variable was included only when supported by sound biological, clinical or epidemiological justification, thereby avoiding redundant or speculative nodes. Secondly, variables were classified according to their expected causal role within the system as exposure, outcome, confounder, mediator or structural determinant, following established frameworks of causal inference [10,11,12]. Thirdly, when a variable could assume multiple roles depending on the analytical context, a conservative classification aimed at estimating the total effect of exposure was prioritized to minimize overfitting and avoid blocking biologically relevant causal pathways. Finally, the use of DAGs was not intended to simplify pathophysiology, but rather to provide a conceptual framework capable of aligning biological interpretation with analytical decision-making, while avoiding decontextualized graphical representations.

### 3.2. Classification of Variables, Causal Roles and Minimal Adjusted Sets

From an epidemiological perspective, this approach is fundamental, as the same factor can play different roles depending on the outcome being assessed and the level of analysis. For example, nutrition can act as a mediator in the relationship between environmental stimuli and milk production by influencing energy balance and the availability of metabolic substrates; however, in behavioral analysis, it can also operate as a structural determinant by conditioning feeding behavior and the organization of grazing time. Similarly, temperament constitutes a baseline trait of the individual that modulates the reactivity of the hypothalamic–pituitary–adrenal axis, simultaneously affecting cortisol levels, HRV and the behavioral response to environmental stimuli, positioning it as a potential effect modifier in HRV and production models [27]. In this regard, the value of DAGs does not lie in reducing biological complexity, but in making explicit analytical decisions underlying the estimation of causal effects. By clarifying the relationships between variables, DAGs enable the identification of pathways of confounding, the avoidance of inappropriate adjustments, and the strengthening of consistency between the statistical model and the pathophysiological plausibility of the cattle production system.

Across the three DAGs, the node “stress” retains the same core biological meaning: an integrated physiological state reflecting activation of stress-regulatory systems, particularly hypothalamic–pituitary–adrenal and autonomic responses, arising from thermal load, pain or disease, handling and movement, social instability, nutritional challenge, or abrupt management changes [4,5,6,7,8,9]. Its causal role, however, is outcome-specific. In the milk production DAG, stress is modeled as an intermediate pathway through which environmental and management conditions can affect neuroendocrine milk ejection and performance; in the behavioral DAG, it links environmental and social challenges to observable behavioral organization; and in the HRV DAG, it is a proximal determinant of autonomic modulation. For estimation of a total music effect, post-exposure stress should generally be treated as a mediator and not adjusted away, whereas stress indicators measured before acoustic exposure may be considered baseline covariates or effect modifiers when they influence both exposure conditions and the outcome. This distinction prevents the single node from being interpreted as biologically different constructs across the three graphs.

## 4. Biological Plausibility of Acoustic Exposure

### 4.1. Environmental Stress and Physiological Regulation

Environmental stress in grazing dairy cattle is cumulative and time-varying rather than a single discrete event. Thermal load, pain and lameness, walking demands, social instability, management changes, and nutritional constraints can converge on HPA and autonomic pathways and alter intake, metabolism, behavior, reproductive function, milk production, and welfare [4,5,6,7,8,9]. Evidence from ruminants shows that high environmental temperature can impair physiological regulation and productive efficiency, while temperature and relative humidity measurably influence peripheral thermal responses [4,5,6,7,8]. An acoustic intervention therefore occurs against a pre-existing and fluctuating physiological load. Music should be modeled as one sensory input within this system, with effects expected to depend on baseline health, environmental conditions, physiological stage, and management rather than as a stand-alone treatment.

### 4.2. Neuroendocrine Modulation and Oxytocin Release

The hypothesis that music can modify a cow’s productive performance or welfare is only tenable if there is a plausible biological pathway between the acoustic signal and the systems that regulate behavior, metabolism and mammary function. This plausibility rests primarily on three axes: the neuroendocrine modulation of milk ejection, the activation or attenuation of the hypothalamic–pituitary–adrenal (HPA) axis, and the regulation of the autonomic nervous system, which can be quantified in part by HRV. Napolitano et al. [16] highlights that milk ejection depends on the pulsatile release of oxytocin and the contraction of myoepithelial cells in the mammary gland. This process, although closely associated with milking, is highly sensitive to the emotional context and handling methods. When the environment promotes safety and predictability, the oxytocinergic response tends to be more efficient. In contrast, aversive stimuli, pain, abrupt changes in routine or sensorially hostile environments elevate HPA activity, increase cortisol and compromise the functional release of oxytocin. Skånberg et al. [28] demonstrated that even a positive tactile stimulus, such as the use of a mechanical brush, increases oxytocin in dry cows; although this does not involve music, the finding is relevant because it supports the physiological principle that positive sensory interventions can modulate the animal’s endocrine environment.

### 4.3. Autonomic Regulation and Heart Rate Variability

The autonomic nervous system (ANS) regulates involuntary functions such as heart rate, vascular tone, thermoregulatory sweating, and pupillary responses through coordinated sympathetic and parasympathetic activity. HRV provides a non-invasive window into this regulation by quantifying variation in successive R-R intervals and is influenced by both sympathetic and vagal modulation [23,24,25,26]. In the context of acoustic exposure, a response may become evident at the autonomic level before it is detectable as a change in milk yield; potential manifestations include altered vagal modulation, sympathetic reactivity, rumination, posture, or vigilance.

HRV should not be interpreted as a simple one-dimensional “balance” score: time- and frequency-domain metrics capture different aspects of cardiac autonomic modulation and are sensitive to respiration, movement, health status, pain, and other contextual factors [23,24,25,26,29,30]. In cattle, HRV has been proposed as a useful non-invasive tool for detecting stress-related changes, while evidence from other mammals supports its use as an indicator of autonomic state and regulatory dysfunction [23,24,25,26,29,30].

## 5. Biological and Causal Determinants of Milk Production

### 5.1. Stress and Neuroendocrine Regulation

In the production DAG, stress is central because it connects environmental conditions, disease, nutrition, management, and neuroendocrine regulation. Rather than repeating a fixed list of stressors, the causal interpretation distinguishes baseline stress burden from changes in stress after acoustic exposure. Pre-existing pain, heat load, or health problems can alter both physiological responsiveness and milk yield, whereas a change in stress after music begins is part of the hypothesized mediating pathway. Therefore, adjustment must be aligned with the estimand: baseline stress indicators may be included when they open confounding pathways, but post-exposure stress should not be controlled when estimating the total effect of music. This distinction is consistent with evidence that stress-related endocrine changes can affect feed intake, immune function, oxytocin-mediated milk ejection, and productive efficiency [9,16,27].

### 5.2. Nutrition and Metabolic Mediation

Nutrition appears in the DAG as a partial mediator between music and production, but this role must be interpreted with precision. From a clinical perspective, nutrition is not merely a matter of quantity of feed, but also of forage quality, energy synchrony, ruminal health and the ability to maintain energy balance. Habimana et al. [4] document that heat stress reduces dry matter intake and alters metabolites linked to production. Camargo et al. [17] add that in tropical and subtropical environments, forage variability determines the expression of productive potential. Vanholder et al. [18] and Melendez and Serrano [19] identify negative energy balance and subclinical ketosis as key mechanisms underlying reduced production and health vulnerability. The causal significance of nutrition is twofold. On the one hand, a musical intervention could improve the emotional atmosphere during milking and, indirectly, reduce behavioral inhibition or promote more stable patterns of feed intake and rumination. On the other hand, this potential effect will only be visible if the system has the biological capacity to respond. In cows with ketosis, hypocalcemia or severe feed intake restriction, music is unlikely to offset the underlying physiological cost. Hence, the Music × Nutrition interaction is one of the strongest hypotheses in the production DAG.

### 5.3. Postpartum Diseases

Disease was incorporated into the production DAG because it simultaneously modifies behavior, stress physiology, feed intake, autonomic activity and productive efficiency. In epidemiological terms, this makes disease a structural confounder rather than a simple secondary variable. Lameness, mastitis and metabolic disease alter resting behavior, movement, rumination time and social interaction. Jurkovich et al. [9] describe how pain and inflammatory processes modify stress physiology and productive performance simultaneously. Likewise, Vanholder et al. [18] show that transition diseases compromise metabolic adaptation and predispose cows to long-term production losses. This means that herds with lower disease burden may appear to respond more favorably to music, even if the observed effect is partially explained by differences in baseline health status. Consequently, disease cannot be interpreted as an isolated pathological condition, but rather as a node capable of reorganizing the entire physiological network represented in the DAG.

### 5.4. Physiological Stage and Reproductive Structure

Physiological stage was incorporated because production capacity and metabolic vulnerability vary substantially throughout lactation. Lim et al. [14] and Temesgen et al. [15] demonstrate that dry period duration, days open and lactation structure influence not only milk yield, but also the susceptibility to additional physiological stressors. Transition cows constitute a particularly sensitive subgroup because endocrine changes, negative energy balance and inflammatory activation occur simultaneously during this period. Therefore, the same acoustic environment may generate different responses depending on lactation stage, metabolic stability and reproductive condition. From a causal perspective, physiological stage acts as both a structural determinant and a potential effect modifier, since it conditions the biological capacity of the animal to respond to environmental stimuli.

### 5.5. Genetic Adaptation and Production Variability

Breed and genetic adaptation were included in the production DAG because productive responses in high-tropical systems are strongly influenced by adaptive capacity to altitude, thermal variability and forage conditions. Camargo et al. [17] describe that productive efficiency in tropical systems depends not only on nutritional management, but also on the interaction between genotype and environment. Likewise, Marçal-Pedroza et al. [27] suggest that behavioral reactivity and endocrine responsiveness vary among animals, reinforcing the possibility of heterogeneous responses to sensory interventions such as music. Consequently, genetic adaptation should not be interpreted merely as a production attribute, but as a biological determinant capable of modifying stress tolerance, metabolic efficiency and behavioral stability within the production system.

## 6. Causal Framework for Behavior

### 6.1. Feeding Behaviour and Resource Access

Behavior in grazing dairy cattle is strongly influenced by the availability and distribution of resources. Access to forage, water and shade determines grazing time, rumination patterns, movement and social interaction. In high-tropical systems, fluctuations in pasture quality and climatic variability may substantially alter behavioral organization. Camargo et al. [17] emphasize that forage variability in tropical environments modifies feeding dynamics and productive adaptation. Likewise, Habimana et al. [4] demonstrate that heat stress alters intake behavior and ruminal activity, thereby affecting both metabolic stability and behavioral expression. Within the behavioral DAG, feeding behavior was therefore interpreted not merely as an outcome, but also as a mediator linking environmental conditions, stress and productive physiology.

### 6.2. Space, Movement, Social Interaction and Grazing Organization

Space availability and movement patterns constitute major structural determinants of bovine behavior in grazing systems. Spatiotemporal tracking studies show that grazing, resting, traveling, resource selection, and herd cohesion vary with grazing management, breed, season, and landscape attributes [20,21]. Walking distance, topography, access routes, water location, and the spatial organization of pasture areas influence energy expenditure, resting time, behavioral synchrony, and resource use. In high-tropical production systems, prolonged movement associated with steep terrain or distant milking facilities may increase physiological load and modify behavioral stability. Consequently, movement should be interpreted as part of the ecological organization of the production system rather than as locomotion alone. The behavioral DAG therefore incorporates space and movement as structural variables capable of conditioning social interaction, feeding patterns, and stress responsiveness. The emotional and social dimensions of cattle behavior are also relevant in grazing systems: Pinelli et al. [22] demonstrated that social interaction and environmental conditions simultaneously influence cortisol, oxytocin, and behavioral responses, while Marçal-Pedroza et al. [27] showed that temperament is associated with endocrine and productive responses. Vigilance, dominance relationships, and group stability may therefore condition the interpretation of behavioral responses associated with acoustic exposure.

### 6.3. Disease, Pain and Behavioral Disruption

Disease and pain directly alter behavioral organization in cattle. Lameness, inflammatory processes and metabolic disorders reduce grazing activity, modify posture, alter rumination and disrupt social interaction. In behavioral terms, the relevant consequence is a contraction of the behavioral repertoire and reduced responsiveness to environmental stimuli, which occurs independently of any acoustic intervention [9]. Consequently, behavioral changes observed during musical exposure could be confounded by underlying disease processes if health status is not adequately considered. Within the behavioral DAG, disease was therefore incorporated as a structural confounder capable of simultaneously influencing stress physiology, movement, feeding behavior and behavioral responsiveness.

### 6.4. Environmental Interpretation of Behavioral Responses

An integrated interpretation of the behavioral DAG indicates that behavioral responses to music are unlikely to depend exclusively on the acoustic stimulus itself. Instead, behavioral expression emerges from the interaction between environmental conditions, stress physiology, disease burden, social organization and management structure. From this perspective, music should be interpreted as a contextual sensory stimulus capable of interacting with broader ecological and physiological processes rather than as an isolated determinant of behavioral change.

## 7. Designed Directed Acyclic Graphs and Causal Structures

### 7.1. DAG for Milk Production

**Figure 1:**
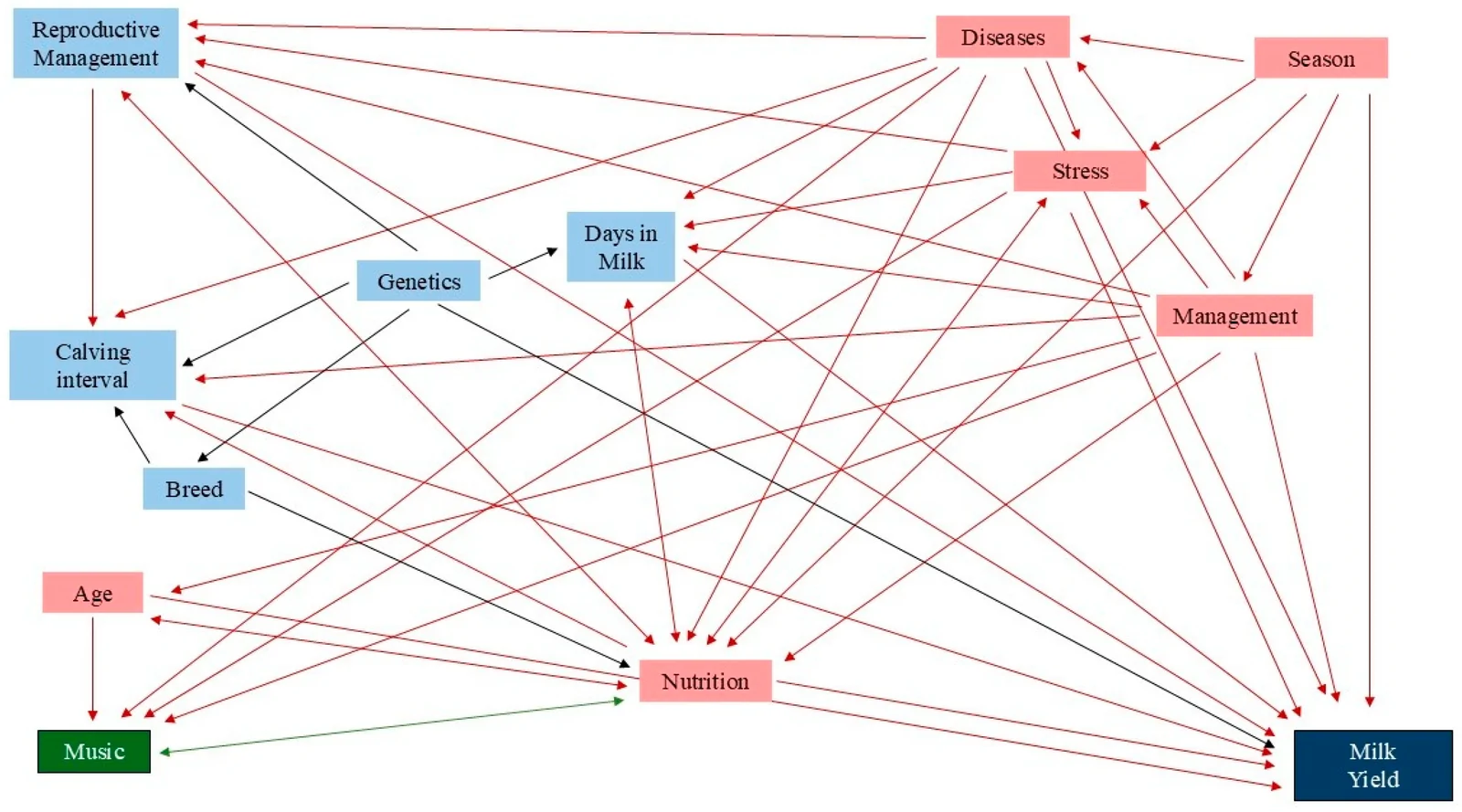
Directed acyclic graph (DAG) represents hypothesized causal relationships between musical exposure and milk yield in high-tropical grazing dairy cattle. Green boxes denote the exposure, dark blue the outcome, light blue structural biological determinants, and pink potential confounders, mediators, or management-related variables. Green arrows show the primary hypothesized music-related pathway, red arrows additional causal pathways, and black arrows structural biological relationships.

The milk production DAG (Figure 1) was constructed to represent the hypothesized causal relationships linking musical exposure with milk yield under high-tropical grazing conditions. Within this structure, music was positioned as the exposure variable, whereas milk production constituted the principal outcome. Stress occupied a central position because it simultaneously connects environmental conditions, disease burden, nutritional status and neuroendocrine regulation. Nutrition was incorporated as a partial mediator because it may influence production through changes in feed intake, ruminal stability and energy balance. However, nutrition may also behave as a structural determinant when forage availability and metabolic adaptation condition the biological capacity of the animal to respond to environmental stimuli. Disease was incorporated as a structural confounder because it simultaneously alters stress physiology, feeding behavior, autonomic regulation and productive efficiency. Likewise, physiological stage, reproductive structure and transition dynamics were included because endocrine adaptation and metabolic vulnerability vary substantially throughout lactation. Breed and genetic adaptation were also incorporated because productive performance in high-tropical systems depends strongly on environmental adaptability, behavioral stability and thermal tolerance. Consequently, the production DAG proposes that any productive effect associated with music is unlikely to occur through direct stimulation of milk synthesis itself, but rather through indirect modulation of stress physiology, environmental predictability and behavioral stability during milking.

### 7.2. DAG for Behaviour

**Figure 2:**
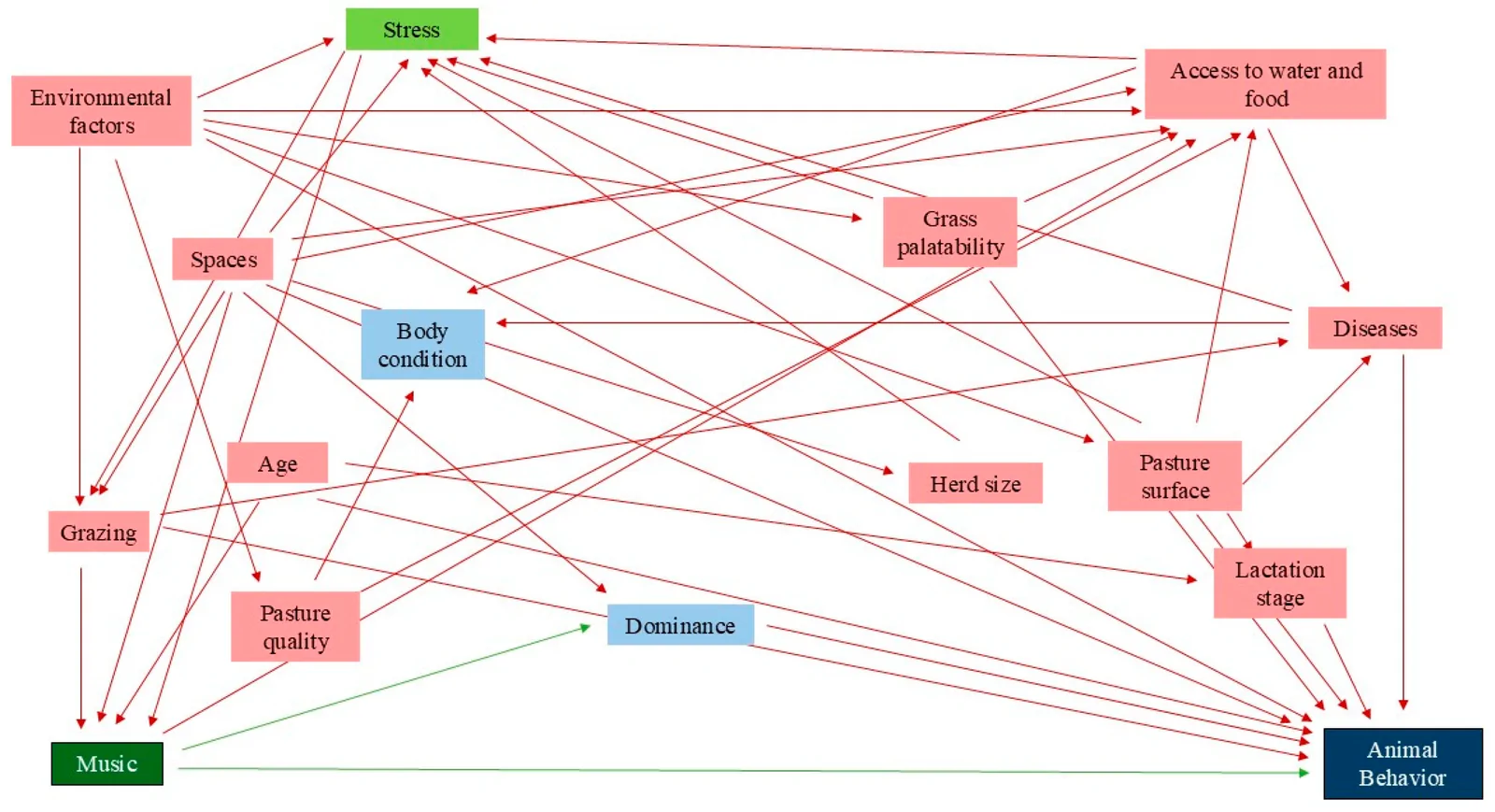
Directed acyclic graph (DAG) represents hypothesized causal relationships between musical exposure and animal behavior in high-tropical grazing dairy cattle. Green boxes denote the exposure, dark blue the outcome, light blue structural biological or social determinants, and pink environmental, physiological, ecological, or confounding variables. Green arrows show the primary hypothesized music-related pathway and red arrows additional causal pathways.

The behavioral DAG (Figure 2) was developed to represent the ecological and physiological determinants capable of modifying behavioral responses associated with musical exposure in grazing dairy cattle systems. Within this framework, behavior was interpreted as a multidimensional outcome influenced simultaneously by environmental conditions, disease status, social organization, feeding dynamics and stress physiology. Consequently, behavioral responses cannot be interpreted independently from the ecological structure in which the animals interact. Feeding behavior and resource access were incorporated because forage availability, water access and pasture organization condition grazing time, rumination and movement patterns. Likewise, space availability, walking distance and topography were included as structural determinants capable of modifying behavioral synchrony and energy expenditure. Disease and pain were incorporated as major confounding variables because of lameness, inflammatory processes, and metabolic instability simultaneously alter posture, locomotion, vigilance behavior, and social interaction. Temperament and social interaction were also considered important modifiers of behavioral responsiveness because emotional reactivity and group stability influence the interpretation of environmental stimuli. Therefore, the behavioral DAG suggests that music should not be interpreted as an isolated determinant of behavioral change, but rather as a contextual sensory stimulus interacting with broader ecological and physiological processes.

### 7.3. DAG for Heart Rate Variability (HRV)

**Figure 3:**
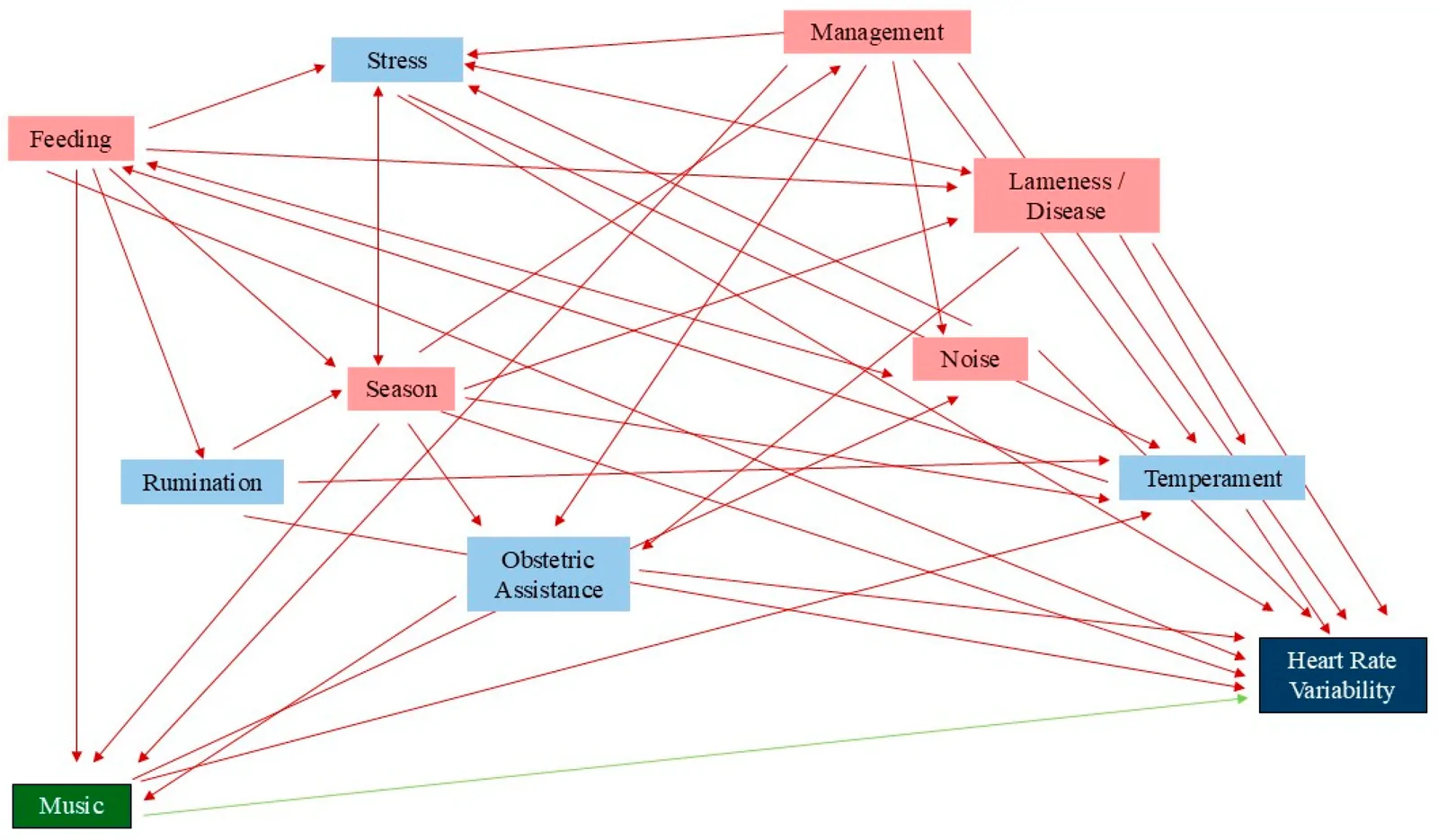
Directed acyclic graph (DAG) representing hypothesized causal relationships between musical exposure and heart rate variability (HRV) in high-tropical grazing dairy cattle. Green boxes denote the exposure, dark blue the outcome, light blue structural biological determinants, and pink physiological, environmental, or confounding variables. Green arrows show the primary hypothesized music-related pathway and red arrows additional causal pathways.

The HRV DAG (Figure 3) was developed to represent the causal architecture linking musical exposure with autonomic regulation in dairy cattle systems. Within this structure, HRV was interpreted as an intermediate physiological outcome reflecting the dynamic balance between sympathetic and parasympathetic autonomic activity. Consequently, HRV responses associated with music were hypothesized to occur primarily through indirect modulation of stress physiology, emotional reactivity and environmental perception. Stress was positioned as a major structural determinant because autonomic balance is highly sensitive to environmental instability, disease burden and management-related stressors. Likewise, disease and pain were incorporated as confounding variables capable of simultaneously modifying cardiovascular regulation, behavioral stability and physiological responsiveness. Temperament was incorporated as an effect modifier because individual autonomic responsiveness may condition the magnitude and direction of HRV responses associated with acoustic exposure. Environmental conditions and management structure were also included because thermal load, movement, milking routine and ecological instability strongly influence baseline autonomic activity in grazing systems. Taken together, the HRV DAG suggests that autonomic responses to music are unlikely to represent isolated cardiovascular effects, but rather components of a broader adaptive response involving stress regulation, environmental perception and behavioral modulation.

### 7.4. Comparative Interpretation of Causal Pathways

A comparative summary of the epidemiological structure, biological domains, confounding pathways, mediators, and expected responses associated with each DAG is presented in Table 1. Comparison of the three DAGs reveals that stress physiology constitutes the principal integrative node linking environmental exposure, behavior, autonomic regulation and productive performance. However, the causal architecture differs substantially according to the outcome under evaluation. In the design of the DAG, nutrition, disease and physiological stage occupy central causal positions because productive responses depend strongly on metabolic adaptation and endocrine stability. In contrast, the behavioral DAG is more strongly structured around ecological organization, social interaction and resource distribution. The HRV DAG, meanwhile, is characterized by a greater influence of autonomic responsiveness, stress physiology and environmental instability. Despite these differences, all three DAGs converge in suggesting that the effects of musical exposure are unlikely to occur independently from broader environmental and management-related conditions. Consequently, causal interpretation of acoustic interventions in dairy cattle systems requires simultaneous consideration of physiology, ecology, disease burden, behavioral organization and autonomic regulation.

## 8. Discussion

### 8.1. Clinical Interpretation for DAG Design

The production DAG reframes the central question from whether music independently increases milk yield to whether a standardized acoustic intervention changes stress-related and neuroendocrine processes under defined physiological conditions. Productive responses are therefore expected to depend on metabolic capacity, health status, lactation stage, and management context. From a clinical perspective, music cannot substitute for nutritional correction, disease control, or thermal management; it can only be evaluated as a complementary environmental intervention. The main epidemiological implication is that baseline health and management variables must be represented explicitly, while intermediate post-exposure mechanisms should not be adjusted away when the target estimand is the total effect. This outcome-specific distinction is a principal advantage of the DAG framework [10,11,12,13].

### 8.2. Behaviour as an Ecological and Adaptive Response

The behavioral DAG reinforces the interpretation that behavior in grazing dairy cattle is fundamentally ecological and context-dependent. Behavioral responses associated with music cannot be interpreted independently from environmental conditions, grazing structure, social hierarchy, disease burden and resource availability. This interpretation is particularly relevant in high-tropical systems, where climatic variability, topography, pasture organization and movement patterns continuously modify behavioral organization. Consequently, music should not be interpreted as an isolated behavioral stimulus, but rather as one component of a broader sensory and environmental context capable of interacting with stress physiology, social interaction and adaptive responses. Pinelli et al. [22] demonstrated that environmental and social conditions simultaneously influence behavioral and neuroendocrine responses, supporting the idea that behavioral adaptation emerges from integrated ecological and physiological processes rather than from isolated sensory stimulation. Similarly, Marçal-Pedroza et al. [27] suggest that temperament and endocrine responsiveness may contribute to heterogeneous behavioral responses among animals exposed to the same environmental conditions. The behavioral framework also suggests that apparent calming or stimulatory effects attributed to music may partially reflect changes in environmental predictability and stress perception rather than direct behavioral conditioning.

### 8.3. HRV as an Intermediate Mechanistic Outcome

Among the three proposed outcomes, HRV may represent a particularly sensitive physiological indicator for evaluating early responses to musical exposure because autonomic modulation can occur over shorter time scales than changes in productive performance. Unlike milk production, which depends on sustained metabolic and endocrine adaptation, HRV can respond rapidly to subtle alterations in autonomic balance and environmental perception. The HRV DAG suggests that autonomic responses associated with music are likely mediated through indirect modulation of stress physiology, emotional reactivity and vagal activity. However, HRV interpretation remains highly vulnerable to confounding by disease, pain, thermal stress, management instability and temperament. Mohr et al. [23], Konold et al. [24], Fernandes and Seara [25] and Sieciński et al. [26] consistently describe HRV as a highly sensitive indicator of autonomic regulation and stress responsiveness. This supports the interpretation that physiological responses to acoustic exposure may become evident at the autonomic level before behavioral or productive changes are clinically detectable. This finding has important methodological implications because it indicates that future studies evaluating music through HRV should prioritize strict environmental and clinical standardization. Otherwise, autonomic changes attributed to music may instead reflect uncontrolled variation in stress burden, physiological instability or management conditions.

### 8.4. Implications of DAG-Based Interpretation in Grazing Dairy Cattle Systems

One of the principal contributions of this review is the integration of biological plausibility with explicit causal modelling. The use of DAGs facilitates transparent representation of the relationships among environmental exposure, stress physiology, disease, management and productive responses, thereby reducing the risk of biologically implausible interpretations. From an epidemiological perspective, the proposed framework demonstrates that the same variable may act as a confounder, mediator or effect modifier depending on the outcome under evaluation and the analytical context [10,11,12]. This is particularly evident for variables such as nutrition, stress and temperament, which simultaneously participate in metabolic regulation, behavioral adaptation and autonomic responsiveness. The DAG-based approach also highlights that causal interpretation of acoustic interventions cannot be separated from the ecological complexity of grazing dairy systems. Consequently, future observational and experimental studies should incorporate explicit causal structures during study design, variable selection and statistical adjustment.

### 8.5. Implications for Future Observational and Experimental Studies

The proposed framework suggests that future studies should standardize both the acoustic intervention and the biological context before estimating effects. Music should be reported as a reproducible exposure, including source material or composition, tempo and rhythm, spectral content, sound-pressure level [dB(A)], speaker location and distance, duration, timing relative to milking or grazing, number of exposures, habituation period, and ambient-noise conditions. Randomized crossover, Latin-square, cluster-randomized, or repeated-measures designs with an appropriate no-music or acoustically matched control should be prioritized, with washout periods when carry-over is plausible. Pre-specified DAGs should guide covariate selection and explicitly distinguish baseline stress from post-exposure stress so that total and mediated effects are not conflated.

Multimodal longitudinal measurements are also needed. Future studies should collect HRV alongside synchronized behavioral observations, milk yield and flow variables, rumination and feeding activity, lameness and disease status, body condition, lactation stage, cortisol or other stress-related biomarkers, and, where feasible, oxytocin-related measures. Environmental data should include temperature, relative humidity, solar radiation, rainfall, pasture quality, walking distance, topography, and ambient sound. Wearable sensors, GPS, and machine-learning classification of grazing behavior can improve temporal alignment between acoustic exposure, movement, resource use, and autonomic responses [20,21]. Multicenter work across breeds, altitude ranges, grazing systems, and seasons is needed to test transportability, dose–response, habituation, individual preference, and whether responses differ among conventional music, species-relevant acoustic material, and non-musical controls.

This review has limitations. It is an analytical narrative review rather than a systematic review or meta-analysis; consequently, it does not provide pooled effect estimates or a formal risk-of-bias assessment. Direct bovine evidence on musical interventions remains limited and heterogeneous, and very few studies jointly evaluate milk production, behavior, and HRV in high-tropical grazing conditions. Several DAG edges therefore rely on mechanistic evidence from related livestock or other mammalian models, and the graphs represent explicit hypotheses rather than empirically validated causal structures. The literature search was completed in March 2026, so subsequent evidence is not represented. These limitations reinforce the need to prospectively test the proposed pathways, compare alternative DAGs, assess unmeasured confounding through sensitivity analyses, and report sufficient exposure and management detail to permit replication.

## 9. Conclusions

Musical exposure in dairy cattle should not be interpreted as an isolated stimulus capable of directly modifying production, behavior or autonomic activity. Instead, its potential effects emerge from complex interactions involving stress physiology, environmental conditions, disease burden, nutritional status, physiological stage and management structure. The proposed DAGs demonstrate that milk production, behavior and HRV possess distinct causal architectures, although all three outcomes converge around stress and environmental modulation as central integrative mechanisms. Among the evaluated outcomes, HRV appears to represent a particularly promising physiological indicator for detecting early responses to acoustic exposure, whereas productive responses are likely to remain highly dependent on metabolic and physiological functionality. The integration of DAG-based causal inference with bovine physiology provides a biologically coherent framework for improving study design, variable selection and epidemiological interpretation in future research evaluating musical interventions in high-tropical grazing dairy cattle.

## Figures and Tables

**Table 1:** Comparative causal structure and epidemiological interpretation of the three proposed DAGs.

Component	Milk Production DAG	Behavior DAG	HRV DAG
Main outcome	Milk yield	Animal behavior	Heart rate variability
Principal biological domain	Metabolic and productive physiology	Behavioral organization	Autonomic regulation
Central integrative node	Stress	Environmental interaction	Autonomic balance
Major confounders	Disease, management, season	Disease, environment, dominance	Disease, baseline stress, noise
Principal mediators	Nutrition, stress	Grazing, access to resources	Stress physiology
Main effect modifiers	Genetics, physiological stage	Dominance, age	Temperament
Environmental influence	High	Very high	High
Physiological sensitivity	Metabolic	Behavioral	Neuroautonomic
Main interpretation	Productive response depends on physiological functionality	Behavior reflects ecological adaptation	HRV reflects autonomic adaptation
Main risk of misinterpretation	Ignoring metabolic confounding	Ignoring ecological structure	Ignoring disease/stress burden
Expected music effect	Indirect and limited	Contextual and adaptive	Early physiological modulation
Biologically plausible pathway	Stress reduction → oxytocin stability → milking efficiency	Reduced stress → behavioral stability	Vagal modulation → autonomic adaptation

DAG directed acyclic graph; HRV, heart rate variability. Confounders, mediators, and effect modifiers are classified according to their hypothesized causal role within each DAG and may vary depending on the outcome under evaluation. Arrows represent hypothesized causal relationships and do not necessarily imply direct biological effects.

## Data Availability

No new data were created or analyzed in this study.

## Abbreviations

The following abbreviations are used in this manuscript:

ANS	Autonomic nervous system
DAG	Directed acyclic graph
HPA	Hypothalamic–pituitary–adrenal
HRV	Heart Rate Variability
R-R	R-R Interval
